# Superhydrophobic/superoleophilic cotton-oil absorbent: preparation and its application in oil/water separation

**DOI:** 10.1039/c8ra05420g

**Published:** 2018-08-28

**Authors:** Na Lv, Xiaoli Wang, Shitao Peng, Lei Luo, Ran Zhou

**Affiliations:** School of Environmental Science & Safety Engineering, Tianjin University of Technology Tianjin 300384 China tjutwxl@163.com; Laboratory of Environmental Protection in Water Transport Engineering, Tianjin Research Institute for Water Transport Engineering, Ministry of Transport Tianjin 300456 China

## Abstract

A superhydrophobic and superoleophilic oil sorbent was prepared by attaching SiO_2_ particles onto a cotton fiber surface by a sol–gel method and subsequent octadecyltrichlorosilane modification. The surface formation was confirmed by Fourier transform infrared spectroscopy, scanning electron microscopy, and an observation of the water behavior on the cotton surface. The sorption capacity of the modified cotton in pure oil and in an oil/water mixture, the oil adsorption and the reusability were investigated. Compared with raw cotton, the as-prepared cotton absorbed different oils rapidly up to in excess of 25–75 g g^−1^ its own weight, and the water adsorption was nearly 0 g g^−1^. The modified cotton fiber could separate oil/water mixtures efficiently through a flowing system. After 10 cycles, the as-prepared cotton was still highly hydrophobic with a 6-times greater adsorption than raw cotton. By a simple modification, a low-cost, high-adsorption and environmentally friendly modified cotton could be prepared that can be considered a promising alternative to organic synthetic fibers to clean up oil spills.

## Abstract 2

A superhydrophobic and superoleophilic oil sorbent was prepared by attaching SiO_2_ particles onto a cotton fiber surface by a sol–gel method and subsequent octadecyltrichlorosilane modification.

## Graphical abstract

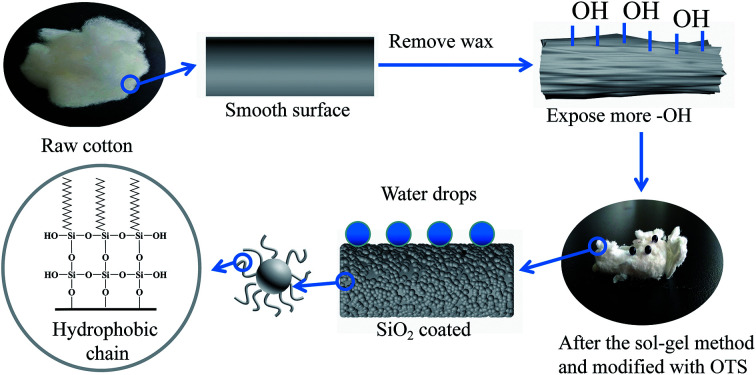

## 1. Introduction

Water pollution by frequent oil spillage has raised major environmental and ecological concerns and has posed threats to human health worldwide.^1^ Recovery by oil-absorbent materials is considered an ideal method to clean oil spills and is applied widely.^2^ Natural organic materials, such as kapok fibers, cattail fibers, sawdust, rice hulls, coconut shells, and bagasse, have become research hotspots because of their rich source, low cost, and biodegradability. They have a good absorptive capacity because of their loose fiber structure and large amount of internal porosity.^3,4^ However, they also have the disadvantage of poor hydrophobicity, and they must be modified before use.^5^ Recently, numerous studies have focused on manufacturing various kinds of hydrophobic materials, and requirements have been achieved mostly by constructing surface-roughness structures and by reducing the surface energy during preparation.^6^ Commonly used methods include the sol–gel method, the electrospinning method, the layer-assembly method, and the sedimentation and hydrothermal methods. Among these methods, the material can be treated by the sol–gel method, and then modified with a low-surface-energy material to make the material hydrophobic. Such a technique would be simple in operation, have a low raw-material price and a high reaction efficiency, and yield a fine rough structure on the material surface.

Cotton is a common crop, with a large output and low production costs, and cotton-fiber products are cheap and often made from multiple fabrics. The cotton fiber is an ideal oil-absorbing substrate because of its small density, and loose internal structure with a large liquid-adsorption space.^7^ Shang *et al.* used ultrasonic irradiation to prepare a silica-containing sol coating on the cotton surface, and the modified sample exhibited good hydrophobicity.^8^ Liang *et al.* adhered polyacrylic acid to the cotton-fabric surface by free-radical polymerization and by virtue of the adhesion ability of dopamine. Ester, the resulting fabric, showed excellent oil–water selectivity.^9^ Dashairya *et al.* used a hydrothermal method and a reduced graphene oxide to build an ultra-thin composite structure on the cotton fibers. The material has a high oil–water separation efficiency and most importantly, after repeated use 10 times, the oil-absorption efficiency still reached 50%.^10^

The main aim of this work was to propose a preparation method for an environmentally friendly and low-cost oil sorbent that can be applied practically in the cleaning and recovery of oil spills on water. The objectives of this study were: (1) to prepare modified cotton fiber that is characterized with regards changes in surface structure and functional groups; (2) to evaluate the adsorption capacities of raw and modified fiber in pure oil and oil/water mixture systems; and (3) to investigate the oil/water separation and reusability of the modified cotton.

## 2. Experimental

### 2.1 Materials

Cotton fiber and peanut oil were from a local market in Tianjin, China. Diesel, lubricating oil, and crude oil were from local gas stations in Tianjin, China. Ammonia, petroleum ether, ethanol, isopropanol, sodium hydroxide (NaOH), tetraethylorthosilicate (TEOS), and anhydrous sodium sulfate (analytical grade) were provided by Tianjin Wind Boat Chemical Reagent Technology Co. Ltd., Tianjin, China. Octadecyltrichlorosilane (OTS, analytical grade) was supplied by Beijing McLean Reagent Ltd., China. Distilled water was produced in the laboratory and artificial seawater was prepared based on a literature ref. 11Table 1 shows the physical properties of the experimental oils at room temperature.

**Table 1:** Physical properties of studied oils at room temperature

Oils	Density (g cm^−3^)	Viscosity (mm^2^ s^−1^)
Diesel oil	0.824	6.62
Peanut oil	0.907	8.5
Lubricating oil	0.893	52
Crude oil	0.912	0.996

### 2.2 Preparation process

Cotton was washed with distilled water three times to remove impurities, boiled in a 3% NaOH solution for 10 min, washed to neutral (pH ∼7), and dried at 80 °C for 4 h.

Ammonia, isopropanol, and deionized water were mixed in a ratio of 6.3 : 40 : 22, and 4 ml TEOS was added dropwise. The pretreated cotton was immersed into a mixture solution and stirred with a magnetic stirrer for 10 min to make it uniform. The solution turned from transparent to turbid and formed a white gum. After standing for 5 h, the fiber was washed with deionized water, and dried at 100 °C. A layer of SiO_2_ particles was attached to the fiber surface after this step.

The altered fiber was immersed in a 2% OTS/anhydrous ethanol mixture for 2 h, removed from the modified solution, washed with ethanol three times to remove the residual solution, and dried at 80 °C.

### 2.3 Characterization

The Fourier transform infrared (FTIR) spectra were characterized on a Tensor 37 spectrometer. The cotton morphologies were obtained by using scanning electron microscopy (SEM, Hitachi-SU3500, Hitachi, Ltd., Tokyo, Japan). Energy-dispersive spectrometry (EDS, Hitachi, Ltd., Tokyo, Japan) was then performed. Before the SEM observation, all cotton surfaces were sputtered with gold by vacuum scanning. Water-contact-angle (CA) measurements were carried out using a DSA 100 (Krüss Company, Ltd., Hamburg, Germany) with a drop of water (3 μL). The residual oil concentrations were analyzed by ultraviolet (UV)-visible (vis) spectroscopy (UV-9600, Beijing General Analysis Instrument Co., Ltd., Beijing, China) at 510 nm. Brunauer, Emmett and Teller (BET) Surface Area was measured by VacPrep 061 (Micromeritics, TrisStra II 3020, Atlanta, USA) at 77 K.

### 2.4 Adsorption and oil–water separation experiments

The sorption capacities of the raw cotton and the cotton that was modified with OTS in pure water and in an oil system were measured by using various liquids (artificial seawater, crude oil, diesel oil, peanut oil, and lubricating oil). The cotton (1 g) was placed into water and immersed in oil at room temperature, and measurements were taken every 5 min. According to ASTMF-726-12, the test measures the rapid adsorption capacity (15 min soaking) and 24 h adsorption capacity. The adsorption capacity is expressed as follows:^12^1
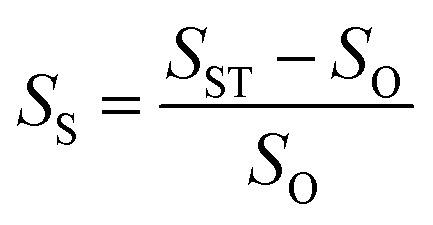
where *S*_S_ is the sorption rate (g (liquid)/g (sorbent)), and *S*_O_ and *S*_ST_ are the quality of the cotton fiber before and after sorption, respectively.

### 2.5 Batch experiments

Diesel was added to 200 ml of artificial seawater in a water-bath shaker. The modified material was placed into the shaker for 15 min at a fixed frequency. The oil-absorbed cotton was removed after 15 min of continuous shaking. The remaining system was studied by UV-vis spectroscopy. The oil-adsorption capacity at equilibrium (*Q*) was calculated from:2
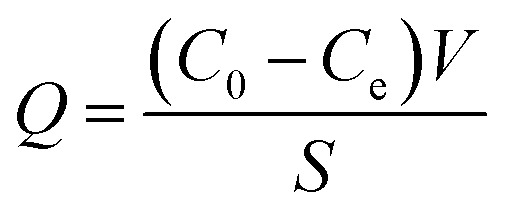
where *C*_0_ is the initial concentration of diesel, *C*_e_ is the equilibrium concentration (g L^−1^) at any time *t*, *V* is the volume of solution (L), and *S* is the adsorbent mass (g). In addition, the separation efficiency (*R*%), could be calculated by the equation:

3 *R*(%) = (1 − *C*_P_/*C*_i_) × 100%

where *C*_P_ is oil content in water after one time separation and *C*_i_ is the oil content in the original oil/water mixture.

### 2.6 Oil-retention performance

A single oil-absorption capacity was tested, and the oil-absorbing material was placed on the filter to drain. After a period of time, the oil-absorption material was weighed and the oil-retention ratio was calculated according to eqn (2).

### 2.7 Reusability

Cotton fiber that absorbed oil from the oil surface was removed by using a mesh screen. It was washed with petroleum ether and dried before weighing. The sorption/desorption cycle was repeated ten times to evaluate the recyclability of the cotton.

## 3. Results and discussion

### 3.1 Characterization

#### 3.1.1 Fabrication of superhydrophobic/superoleophilic cotton-fiber surface and oil-sorption mechanism

**Fig. 1:**
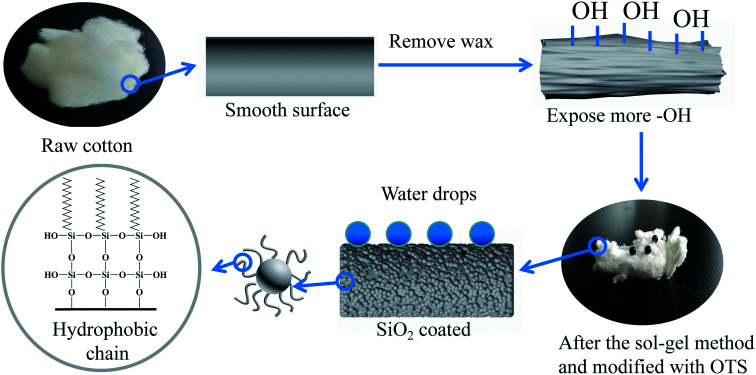
Schematic illustration of the modification cotton fiber.

Fig. 1 shows a schematic illustration of the fabrication process of SiO_2_ particles on the surface of the modified cotton fiber *via* the sol–gel method. Because of the coverage of a small amount of plant wax on the surface of the raw cotton fiber, it is difficult to absorb more oil. The primary function of the NaOH pretreatment is to remove plant wax and to improve the interface bonding properties. After coating the SiO_2_ particles, a rough fiber surface structure was created. The material was immersed in OTS/anhydrous ethanol solution. The hydroxyl groups reacted with the SiO_2_, and long-chain hydrophobic alkyls were introduced onto the cotton-fiber surface with a low surface energy that makes the fiber resistant to wetting by water droplets.^13^

#### 3.1.2 FTIR spectra and BET analysis

**Fig. 2:**
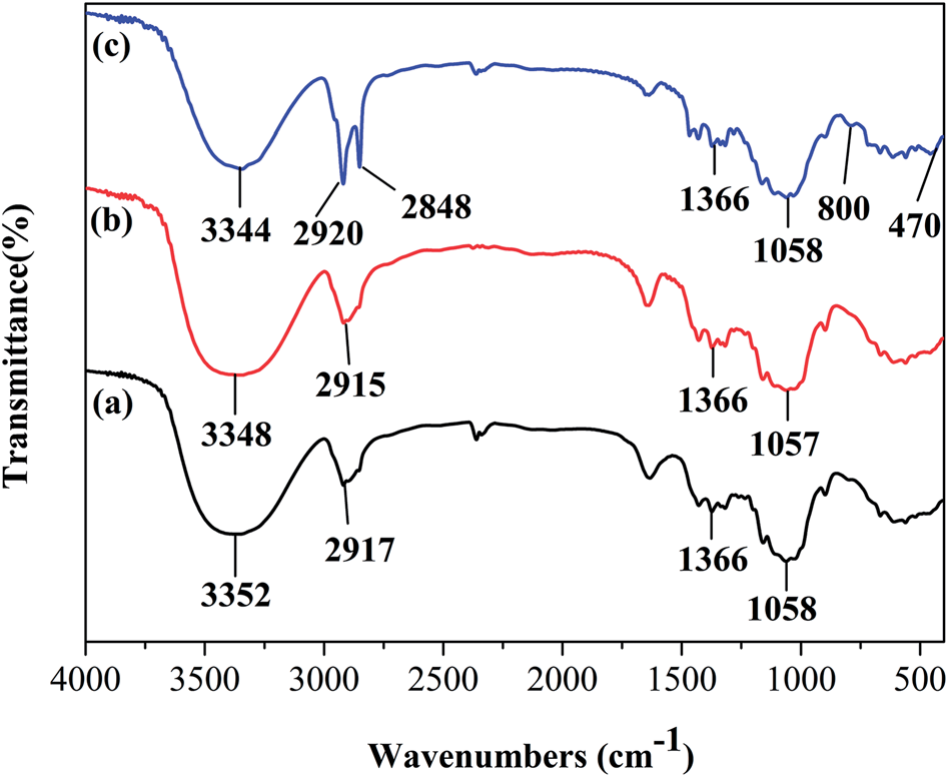
FTIR spectra of (a) raw; (b) SiO_2_ coated and (c) modified cotton fiber.

Fig. 2 shows the FTIR spectra of the raw cotton, the SiO_2_ coated cotton, and the modified cotton. The cotton shows typical characteristic peaks at ∼1058, 1366, 2917, and 3352 cm^−1^, which are assigned to the stretching vibration of C–O, the bending vibration of C–H, the stretching vibration of –CH_2_–, and the stretching vibration of –OH, respectively (Fig. 2a).^14,15^ After the sol–gel method and SiO_2_ coating, there is no obvious change in adsorption peaks (Fig. 2b). In the FTIR spectrum of the modified cotton (Fig. 2c), a sharp peak at 470 and 800 cm^−1^, which is assigned to Si–O–Si groups can be observed, and which indicates that SiO_2_ was coated on the sponge.^16,17^ In the high-frequency region, two peaks at 2848 and 2920 cm^−1^, from CH_2_ and CH_3_ and that were attributed to a long-chain alkyl group that was introduced on the fiber surface, indicated that OTS and cotton fiber were bonded successfully. The intensity of –OH stretching at 3344 cm^−1^ of the modified cotton decreased, which suggests that the hydrophilic groups were replaced mostly by hydrophobic groups.^18,19^

In order to explain the areas change of cotton before and after modification, the total surface areas of the cotton were measured based on the BET model. The specific surface area of the modified cotton were 8.49 ± 0.01 m^2^ g^−1^ while raw cotton were 0.04 ± 0.16 m^2^ g^−1^, increased specific surface area contribute to the SiO_2_ particles attached to the surface, and a decent surface area provides better exposure and higher adsorption potential.^20^

#### 3.1.3 Morphology and EDS analysis

**Fig. 3:**
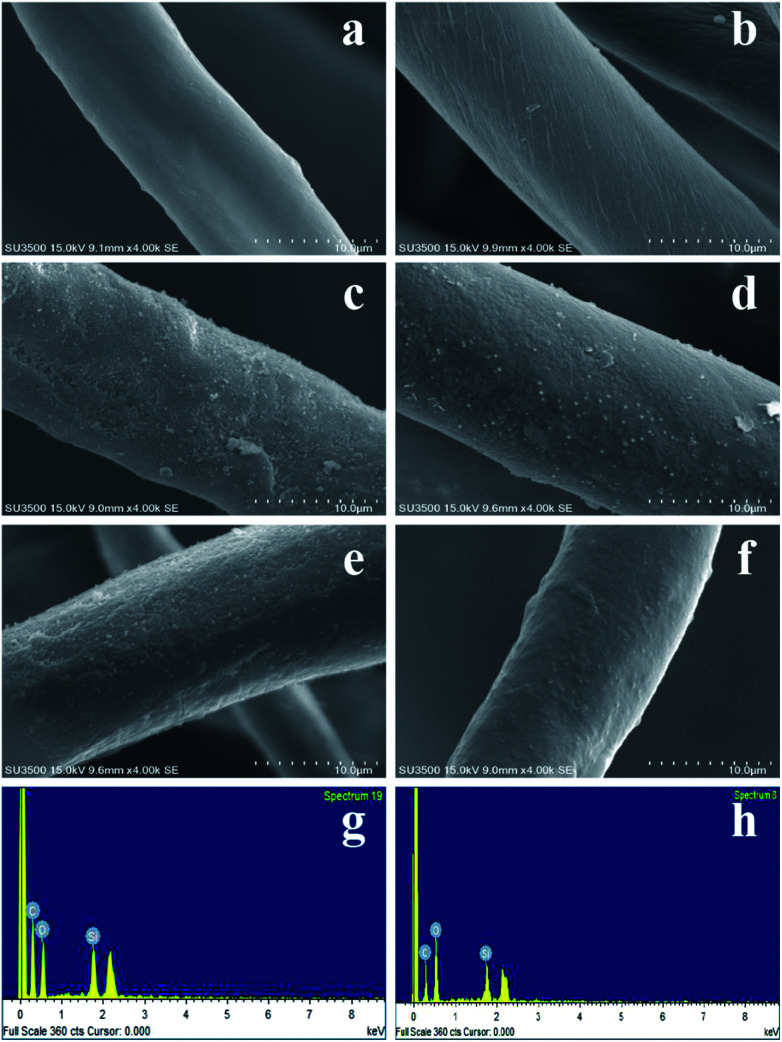
SEM micrographs of (a) raw cotton, (b) pretreated cotton, (c) SiO_2_ coated cotton, (d) modified cotton, (e) absorb oil one time, (f) absorb oil ten times and EDS of SiO_2_ particles change before and after oil absorbed (g and h).

The surface appearances of the raw and modified cotton were investigated by SEM as shown in Fig. 3. The surface of the raw cotton was smooth because of its inherent plant wax (Fig. 3a). After alkaline pretreatment of the raw cotton (Fig. 3b), many subtle textures and wrinkles appeared on the fiber surface, which exposed more hydroxyl ions to attach easily to the SiO_2_ particles. Fig. 3c shows that the fiber surface was covered by SiO_2_ particles and after modification by OTS, SiO_2_ particles attached to the surface of the material evenly and roughened the cotton surface strongly (Fig. 3d). ^15^After absorbed oil one time and ten times (Fig. 3e and f), there was no obviously reduced of SiO_2_ particles on the modified cotton surface. And this result was also demonstrated by EDS analysis (Fig. 3g and h).

#### 3.1.4 Contact angle

**Fig. 4:**
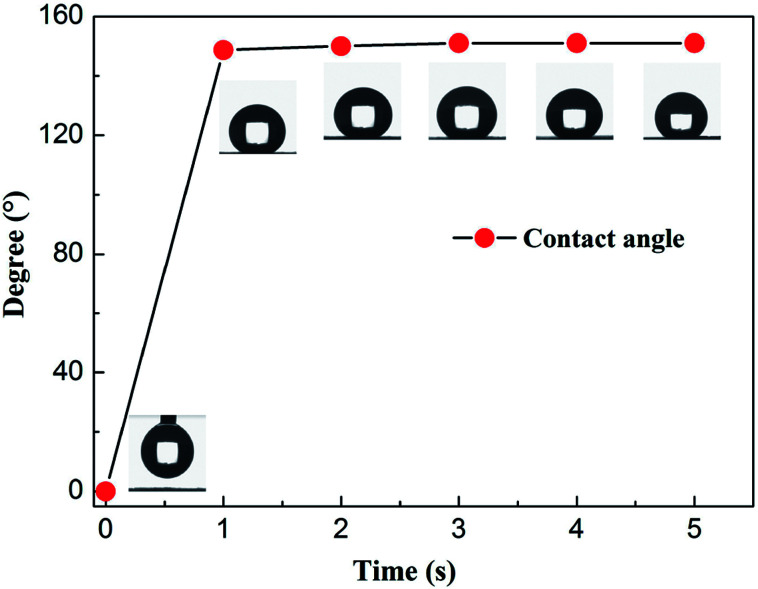
Contact angles change of different time.

In order to clearify the surface wettability of raw and modified cotton, the contact angle was investigated. The water droplet was immediately absorbed by raw cotton, while the water droplet have no changed nearly on the surface of modified cotton (Fig. 7a and b). Moreover, the contact angles of modified cotton of water were 151° ± 1.2° (Fig. 4), indicating the modified cotton have excellent lipophilic and hydrophobicity features.

### 3.2 Analysis of oil adsorption

**Fig. 5:**
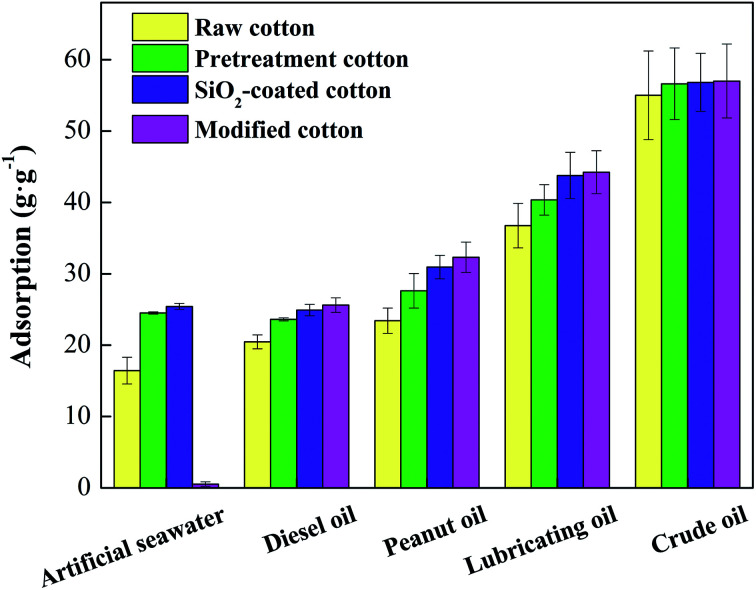
Adsorption capacity of cotton fiber at different stages.

The adsorption capacity before and after modification of the fiber with artificial seawater and oils is shown in Fig. 5. After modification, the water-absorption capacity of the material was reduced significantly and was close to zero. After pretreatment, the water absorption of the cotton increased significantly, because of the removal of wax. A large number of hydroxyl groups in cellulose were exposed and the hydrophilic property of the material increased. The sorption ratios of various oils increased, which indicates that the removal of surface waxes can also increase oil absorption. After SiO_2_ particle attachment and modification with OTS, the adsorption capacities of the modified cotton for diesel, peanut oil, lubricating oil, and crude oil were 25.61 ± 1.02 g g^−1^, 32.32 ± 2.12 g g^−1^, 44.24 ± 3.01 g g^−1^ and 57.01 ± 5.18 g g^−1^, respectively. The adsorption ratio is proportional to the oil density and viscosity.

Table 2 shows the maximum adsorption capacity of the modified cotton fiber compared with other recently reported literature.^18,21–25^ The maximum adsorption capacity of the modified cotton is higher than most of the adsorbents. Some adsorbents had a better adsorption capacity than the modified cotton, but they had a high cost of production and were difficult to biodegrade.

**Table 2:** Comparative adsorption capacities of various adsorbents for oil adsorption

Absorbent	Type of oil	Adsorption capacity (g g^−1^)	References
Modified jute fiber *via* the sol–gel method	Crude oil	7.41	18
	Diesel oil	10.29	
	Lubrication oil	8.48	
Mesoporous silica aerogel	Petrol oil	19.1	20
	Diesel oil	18.6	
The elastic cellulose-based aerogels	Crude oil	77.08	21
	Diesel oil	91.82	
	Lubrication oil	79.79	
Cotton modified using P-SiO_2_ nanoparticles	Diesel oil	20	22
Kapok modified using P-SiO_2_ nanoparticles	Diesel oil	23	
Polyurethane sponge coated poly-TiO_2_	Diesel oil	18	23
	Pump oil	20	
Treated bark	Diesel oil	2	24
Cotton fiber modified *via* the sol–gel method	Diesel oil	25.61	This study
	Lubrication oil	44.24	
	Crude oil	57.01	

**Fig. 6:**
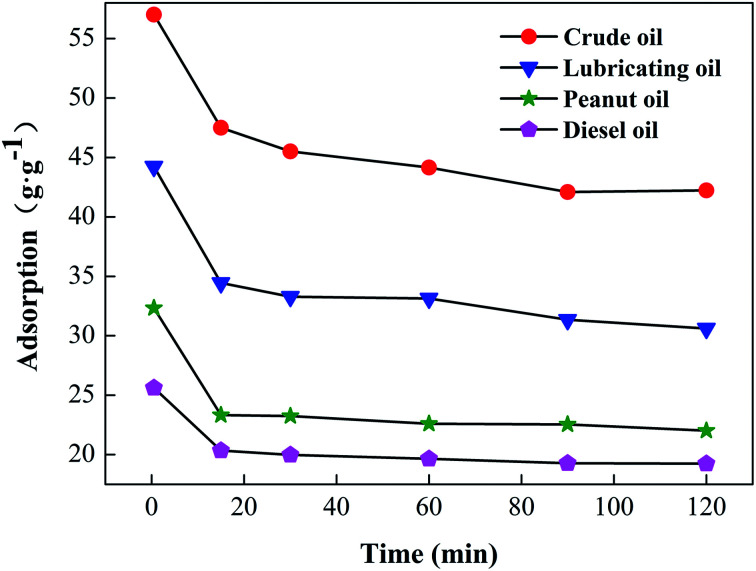
Oil retention of cotton fiber.

The oil-retention performance of the modified cotton was tested in the laboratory by gravity tests. The results are shown in Fig. 6. The loss of oil in the first 15 min was relatively large. Compared with the initial measurements, the absorbability of diesel oil, peanut oil, lubricating oil, and crude oil by the modified cotton was only 83.32%, 77.87%, 72.15%, and 79.42% at 15 min, because, during the adsorption process, some oil adhered to the material surface and became detached after prolonged gravitational action. Lubricating oil and crude oil suffered more loss because of a greater viscosity and density. After 15 min, the decrease in adsorption rate of the modified cotton was gentle, and the oil-retention rate could still reach 70% of the original oil absorption rate after 120 min.

### 3.3 Recyclability

**Fig. 7:**
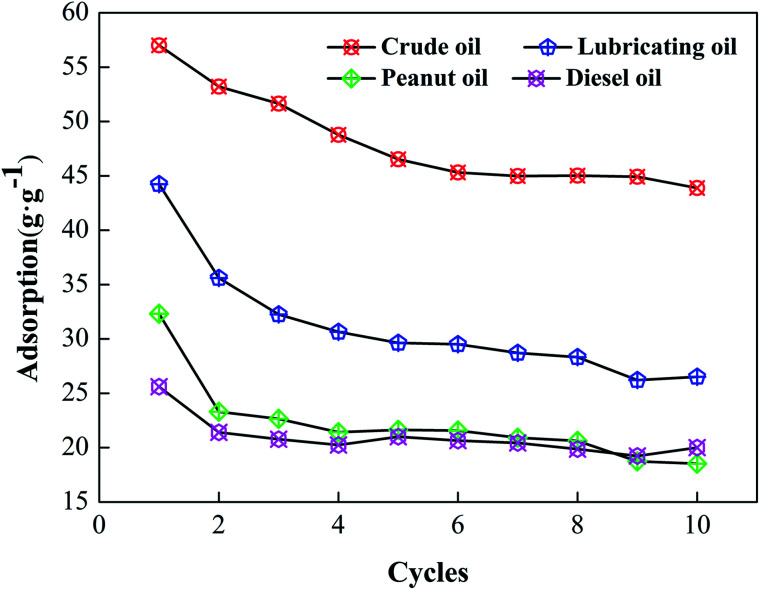
Reusability of modified cotton fiber.

The recyclability of the cotton and the recoverability of the adsorbed oil are important criteria in selecting suitable sorbents for practical applications. To recover the absorbed oil and to reuse the modified cotton, squeezing was selected to verify the sample recyclability. The result is shown in Fig. 7. During the first five cycles of material recycling, the rate of decline in the material is greatest, with the most significant reduction occurring during the first iteration. This occurs because some of the oil is still present on the material surface during the extrusion process. In addition, cavities exist inside the cotton, and it is not easy to discharge these after oil adsorption.^34^

During the entire cycle of reuse, the extrusion force destroys part of the cotton structure, which reduces the adsorption space. However, after 10 times reuse, the adsorption rate of the cotton fiber for different oils exceeded 60%.

### 3.4 Application in water/oil separation

**Fig. 8:**
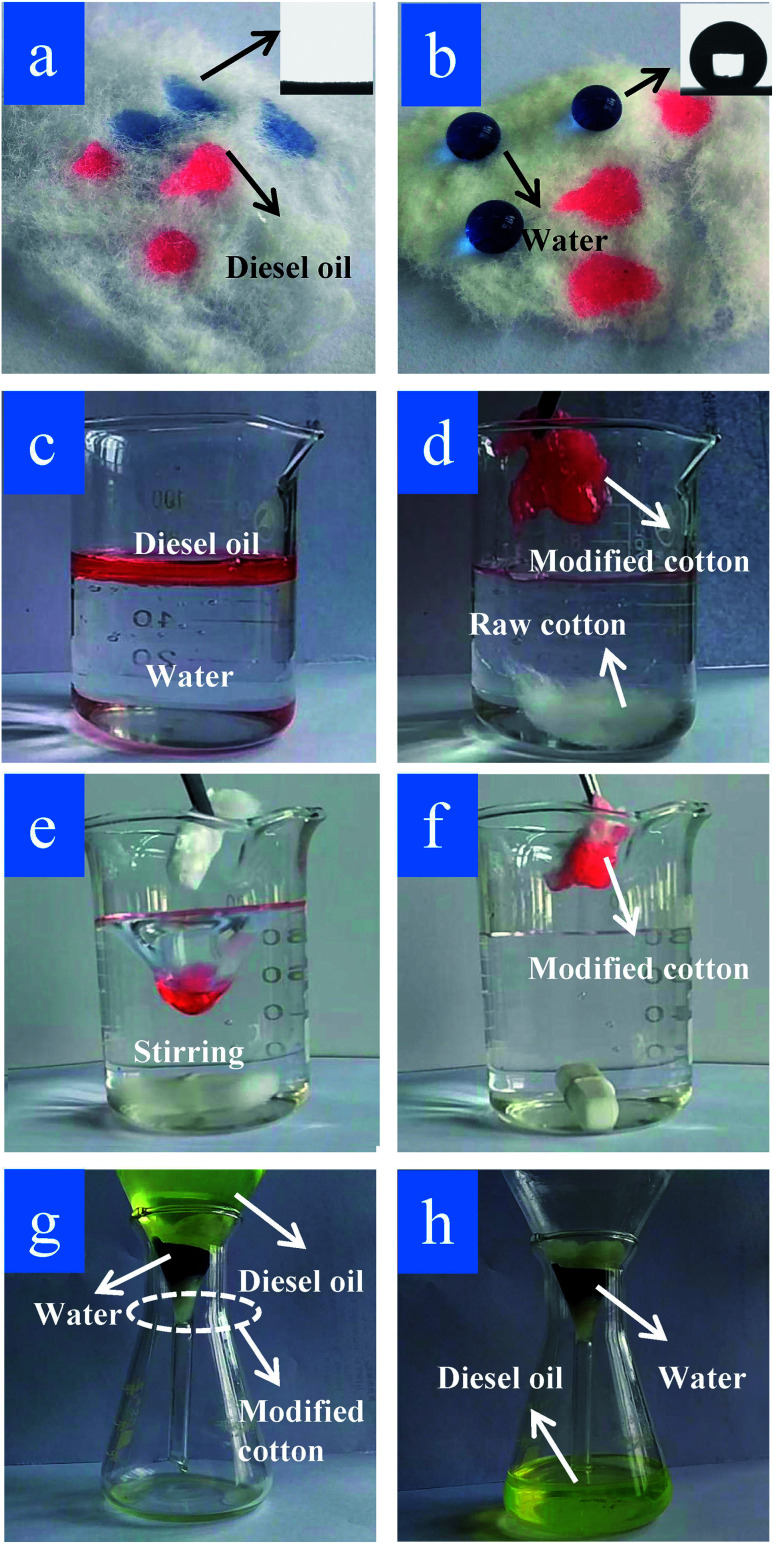
Photographs for clean up of diesel oil from water and oil/water separation. Water droplets (dyed with blue ink) and diesel droplets (dyed with red oil) of (a) raw cotton and (b) modified cotton. (c) Diesel oil floating on the water surface, (d) raw cotton sunken in water and oil was adsorbed by modified cotton. (e) and (f) A vortex-stirring process was used to absorb the diesel oil using modified cotton. (g) and (h) An oil/water mixture was separated by modified cotton.

To exhibit the oil-sorption characteristics of the modified cotton fiber on artificial sea water, an oil-absorption experiment with raw and modified cotton was conducted using the same parameters shown in Fig. 8. As shown in Fig. 8a, the contact angle of the artificial seawater and diesel oil droplets on the raw cotton surface was 0°, while that of the water droplets and the modified cotton was almost 151° ± 1.2°, and the oil droplets were absorbed completely (Fig. 8b). According to Fig. 8c, diesel oil was added into water and floated on the water. When raw and modified cotton were added into a beaker with artificial seawater and diesel, the raw fiber sank and the diesel was absorbed by the modified fiber (Fig. 8d). Furthermore, the separation efficiency of the modified cotton for diesel oil/water mixtures is above 98.5%.

To verify the modified cotton's oil/water separation, Fig. 8e shows a mixture of the oil and water being stirred, and mimics the effect of ocean waves.^19^ After modified-cotton addition, the oil was adsorbed completely, as shown in Fig. 8f. Fig. 8g also shows the modified cotton's separation effect. The modified cotton spread to the bottom of the funnel and was poured into artificial seawater (ink dyeing), so that the water surface was lower than the upper end of the cotton. After standing for 30 minutes, no change occurred and diesel was poured on the water surface. The cotton began to absorb the diesel. Under the effect of gravity, the diesel moved through the cotton and dropped into the funnel. After a period of time, the diesel oil was separated completely and the artificial seawater remained in the water, as shown in Fig. 8h. The results show that the modified cotton has excellent oil/water selectivity and hydrophobicity and has been applied in the field of oil/water separation. Besides, the oil–water separation efficiency of modified cotton in this study was compared with other reported and shown in Table 3, and the results are encouraging.^26–33^ Although there were more composite materials have higher contact angles and oil–water separation efficiency, but cotton have better environmental friendly character and its separation efficiency was also well.

**Table 3:** Comparative oil–water separation capacities of various materials

Materials of oil–water separation	Contact angle	Separation efficiency	References
Spray waste potato residue powders (PRP) and waterborne polyurethane (PU) mixtures on a stainless steel mesh	152° ± 1.3°	96.5%	26
Superhydrophobic polyurethane (PU) sponge is fabricated by coating superhydrophobic attapulgite (APT)	160°	99.87%	27
The superhydrophobic filling bag filled with PU sponges	158° ± 1°	98.2%	28
Nanocellulose sponge	160°	100%	29
Metallic fiber felts	154° ± 2.2°	99%	30
Spray the polytetrafluoroethylene aqueous dispersion onto the cotton fabric	150°	97%	31
Cellulose nanocrystal coated cotton fabric	157°	98%	32
*In situ* growth ZnO nanowires on the surface of carbon fibers	145.4°	98%	33
Modified cotton	151° ± 1.2°	98.5%	This study

### 3.5 Effect of different factors on adsorption and diesel oil movement

**Fig. 9:**
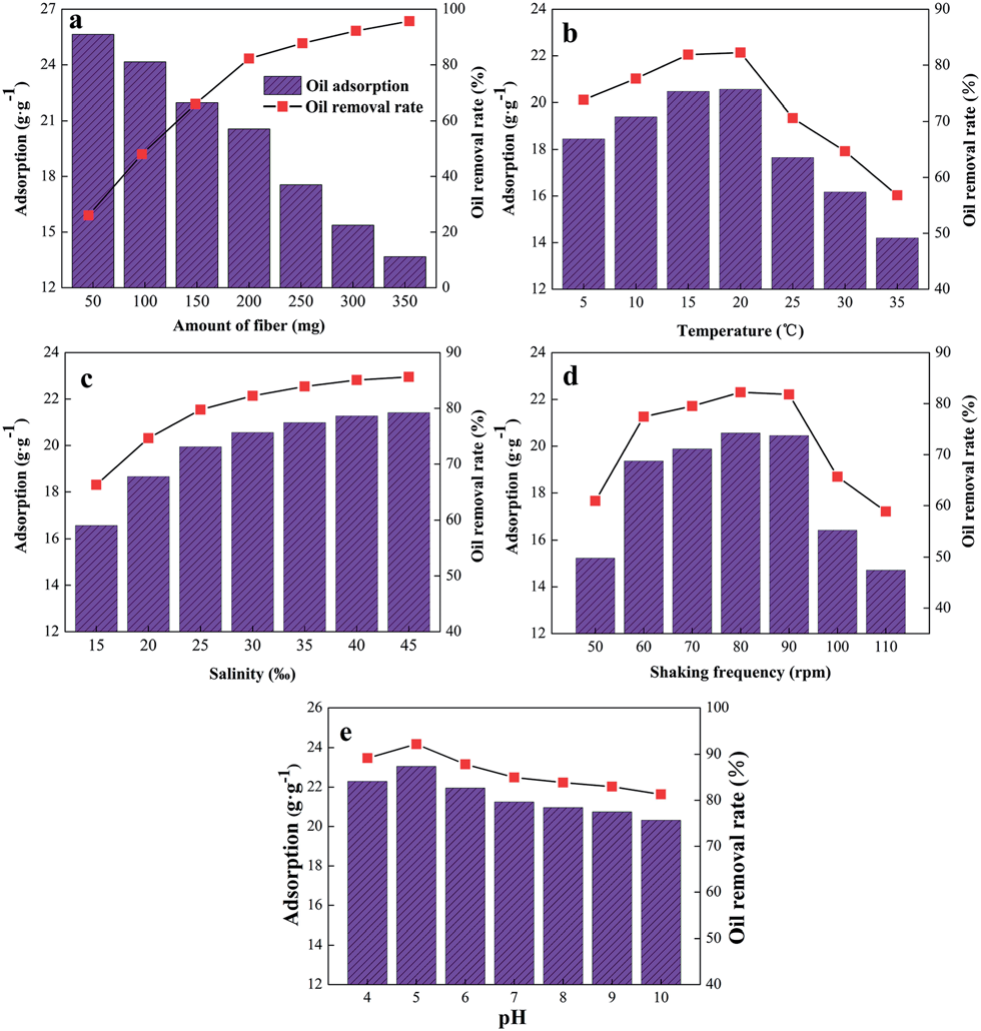
Effect of different factors on adsorption and oil moving rate of modified fibers (a) amount of fiber; (b) temperature; (c) salinity, (d) oscillation frequency and (e) pH.

To use the oil absorbed efficiently, the effect of different factors on the cotton fiber adsorption was tested, as shown in Fig. 9.

#### 3.5.1 Effect of amount of fibers

Artificial seawater (200 ml) with a salt content of 30‰ was added to 5 ml of diesel oil. A beaker was placed in a water-bath shaker at 80 rpm and 20 °C. The adsorption and diesel-oil movement were studied as a factor of the amount of fiber (50, 100, 150, 200, 250, 300, 350 mg) after 15 min. Fig. 9a shows that the oil-absorption rate of the cotton is inversely proportional to the quantity, whereas the oil-removal rate is proportional to the quantity. This observation is consistent with the results of PENG *et al.*^35^ For a fixed amount of diesel, with an increase in the amount of modified cotton added, the contact between the unit mass of cotton and diesel decreased, and the gathered cotton affected the adsorption, which resulted in a decrease in cotton-absorption rate.^36^ Similarly, with an increase in the amount of modified cotton, the contact between the fiber and the diesel oil increases the oil removal rate, but when the quantity exceeds 200 mg, there is little change in the oil-removal rate.

#### 3.5.2 Effect of temperature

To test the impact of quality, a cotton mass of 200 mg was added to artificial seawater at 5, 10, 15, 20, 25, 30, and 35 °C for 15 min, with the results shown in Fig. 9b. The oil-absorption rate and the diesel-oil removal rate increased initially and then decreased with an increase in temperature. When the temperature is lower than 20 °C, the viscosity of the diesel oil is reduced, and it is adsorbed more easily on the cotton surface. At the same time, the temperature is increased. The expansion of cotton fiber increases the adsorption space. When the temperature exceeds 20 °C, the adsorption capacity and oil-removal rate decrease significantly. The reason for this behaviour is that too high a temperature leads to weakened hydrophobic properties of diesel oil, and desorption results. The increasing temperature will accelerate the movement of oil particles and cause difficulties in adsorption.

#### 3.5.3 Effect of salinity

A cotton mass of 200 mg at 20 °C was added to solutions with different salt contents from 15‰ to 45‰. Fig. 9c shows the test results. The oil absorption and diesel-oil removal rate tend to increase with increasing salinity. The main reason for this behaviour is that, as the salt content in the environment increases, a “salting-out” phenomenon occurs, which increases the hydrophobic properties of the oil particles, and makes them easier to adsorb.

#### 3.5.4 Effect of fluctuation

The effect of fluctuation on the adsorption and oil-removal rate with 200 mg cotton, 20 °C and 30‰ salinity was studied at a shaking frequency of 50, 60, 70, 80, 90, 100, and 110 rpm. The results are shown in Fig. 9d. When the shaking frequency was less than 60 rpm and faster than 90 rpm, the fiber adsorption and oil-removal rate were low. When the shaking frequency increased from 50 to 90 rpm, both increased slowly. Within a certain range, the fiber-absorption capacity is proportional to the shaking frequency, with the main reason being the increase in contact rate between the oil and the cotton. When the shaking frequency exceeded the range, the adsorbed oil desorbed, which is not conducive to oil recovery.^37^

#### 3.5.5 Effect of pH

For an efficient oil sorbent, the pH will be able to effect the adsorption rate of oil spill on water surface. The relationship between the pH and sorption capacity of modified cotton is displayed in Fig. 9e. Obviously, the change of pH does not have much influence on the adsorption of oil on modified cotton, and the pH value in the ocean is about 7.7–8.1,^38^ which has little effect on the adsorption performance of the modified cotton.

## 4. Conclusions

A novel method based on cotton fiber and using the sol–gel method was used to prepare superhydrophobic/oleophilic fibers for oil adsorption and oil–water separation. After modification, the absorbed cotton fiber with a roughness and low surface energy imparted the fiber with a better oil/water separation ability and high oil adsorption. The modified cotton could absorb the oil from a mixture of oil/water selectively and exhibited good reusability after several times. The modified cotton was an excellent absorbent owing to its high oil adsorption, low cost, environmental friendliness, excellent recyclability and easy fabrication. These advantages indicate that the modified cotton can be applied in the large-scale cleaning of oil spills on water surfaces.

## Conflicts of interest

There are no conflicts to declare.

## Supplementary Material

## References

[cit1] Guo W. J. (2017). Environ. Pollut..

[cit2] Pinto J., Athanassiou A., Fragouli D. (2018). J. Environ. Manage..

[cit3] Doshi B., Sillanpaa M., Kalliola S. (2018). Water Res..

[cit4] Bazargan A., Tan J., Hui C. W., McKay G. (2014). Cellulose.

[cit5] Maleki H. (2016). Chem. Eng. J..

[cit6] Ma M., Hill R. M. (2006). Curr. Opin. Colloid Interface Sci..

[cit7] Higa N., Nishihama S., Yoshizuka K. (2015). Solvent Extr. Res. Dev., Jpn..

[cit8] Shang Q., Liu C., Zhou Y. (2017). J. Coat. Technol. Res..

[cit9] Liang L., Su M., Zheng C., Li J., Zhan H., Li X., Meng X. (2017). Fibers Polym..

[cit10] Dashairya L., Rout M., Saha P. (2017). Adv. Compos. Mater..

[cit11] Li Y., Gong H., Cheng H., Wang L., Bao M. (2017). Mar. Pollut. Bull..

[cit12] Equipment S, Products M Standard Test Method for Performance of Griddles 1, Annual book of ASTM standards, ASTM International, 2005, vol. 5, pp. 1–17

[cit13] Wang J., Zheng Y., Kang Y., Wang A. (2013). Chem. Eng. J..

[cit14] Wang J., Wang H. (2017). Mar. Pollut. Bull..

[cit15] Liu F., Ma M., Zang D., Gao Z., Wang C. (2014). Carbohydr. Polym..

[cit16] Hsieh C.-T., Chen W.-Y., Wu F.-L., Hung W.-M. (2010). Diamond Relat. Mater..

[cit17] Vinogradova E., Estrada M., Moreno A. (2006). J. Colloid Interface Sci..

[cit18] Lv N., Wang X., Peng S., Zhang H., Luo L. (2018). Int. J. Environ. Res. Public Health.

[cit19] Wang J., Zheng Y., Wang A. (2012). Chem. Eng. J..

[cit20] Kumar A., Kumar A., Sharma G., Al-Muhase A. H., Naushad M., Ghfar A. A. (2018). et al.. Chem. Eng. J..

[cit21] Dai C., Zhang H., Peng S., Wei X., Hu Y. (2017). Mar. Pollut. Bull..

[cit22] Lee J. H., Kim D. H., Han S. W., Kim B. R., Park E. J., Jeong M.-G., Kim J. H., Kim Y. D. (2016). Chem. Eng. J..

[cit23] Tian Y., Zhang X., Liu X., Wang C. (2017). Mar. Pollut. Bull..

[cit24] Shuai Q., Yang X., Luo Y., Tang H., Luo X., Tan Y., Ma M. (2015). Mater. Chem. Phys..

[cit25] Haussard M., Gaballah I., Kanari N., De D. P., Barrès O., Villieras F. (2003). Water Res..

[cit26] Li J., Li D., Yang Y., Li J., Zha F., Lei Z. Q. (2015). Green Chem..

[cit27] Li J., Xu C., Zhang Y., Wang R., Zha F., She H. (2016). J. Mater. Chem. A.

[cit28] Li J., Yan L., Tang X., Feng H., Hu D., Zha F. (2016). Adv. Mater. Interfaces.

[cit29] Phanthong P., Reubroycharoen P., Kongparakul S., Samart C., Wang Z., Hao X. (2018). et al.. Carbohydr. Polym..

[cit30] Zhu H., Guo P., Shang Z., Yu X., Zhang Y. (2018). Appl. Surf. Sci..

[cit31] Lei S., Shi Z., Ou J., Wang F., Xue M., Li W. (2017). et al.. Colloids Surf., A.

[cit32] Cheng Q., Guan C., Wang M., Li Y., Zeng J. (2018). Carbohydr. Polym..

[cit33] Yue X., Zhang T., Yang D., Qiu F., Zhu Y., Fang J. (2018). J. Ind. Eng. Chem..

[cit34] Lim T.-T., Huang X. (2007). Ind. Crops Prod..

[cit35] Peng D., Lan Z., Guo C., Yang C., Dang Z. (2013). Bioresour. Technol..

[cit36] Sidik S. M., Jalil A. A., Triwahyono S., Adam S. H., Satar M. A. H., Hameed B. H. (2012). Chem. Eng. J..

[cit37] Larsen T., Kjeldsen P., Christensen T. H. (1992). Chemosphere.

[cit38] García E., Clemente S., Hernández J. C. (2018). Mar. Environ. Res..

